# Supplementary material for: Inference of multi-enhancer interactions in T lymphocytes using Hi-Cociety
Source: bioRxiv. 2025 Jun 17:2025.06.12.659372. Preprint. [Version 1] doi: 10.1101/2025.06.12.659372 (PMC12262278; doi:10.1101/2025.06.12.659372)
Supplement: 1 [file NIHPP2025.06.12.659372V1-supplement-1.pdf]

bioRxiv preprint doi: https://doi.org/10.1101/2025.06.12.659372; this version posted June 17, 2025. The copyright holder for this preprint (which was not certified by peer review) is the author/funder, who has granted bioRxiv a license to display the preprint in perpetuity. It is made available under aCC-BY 4.0 International license.

Figure S1

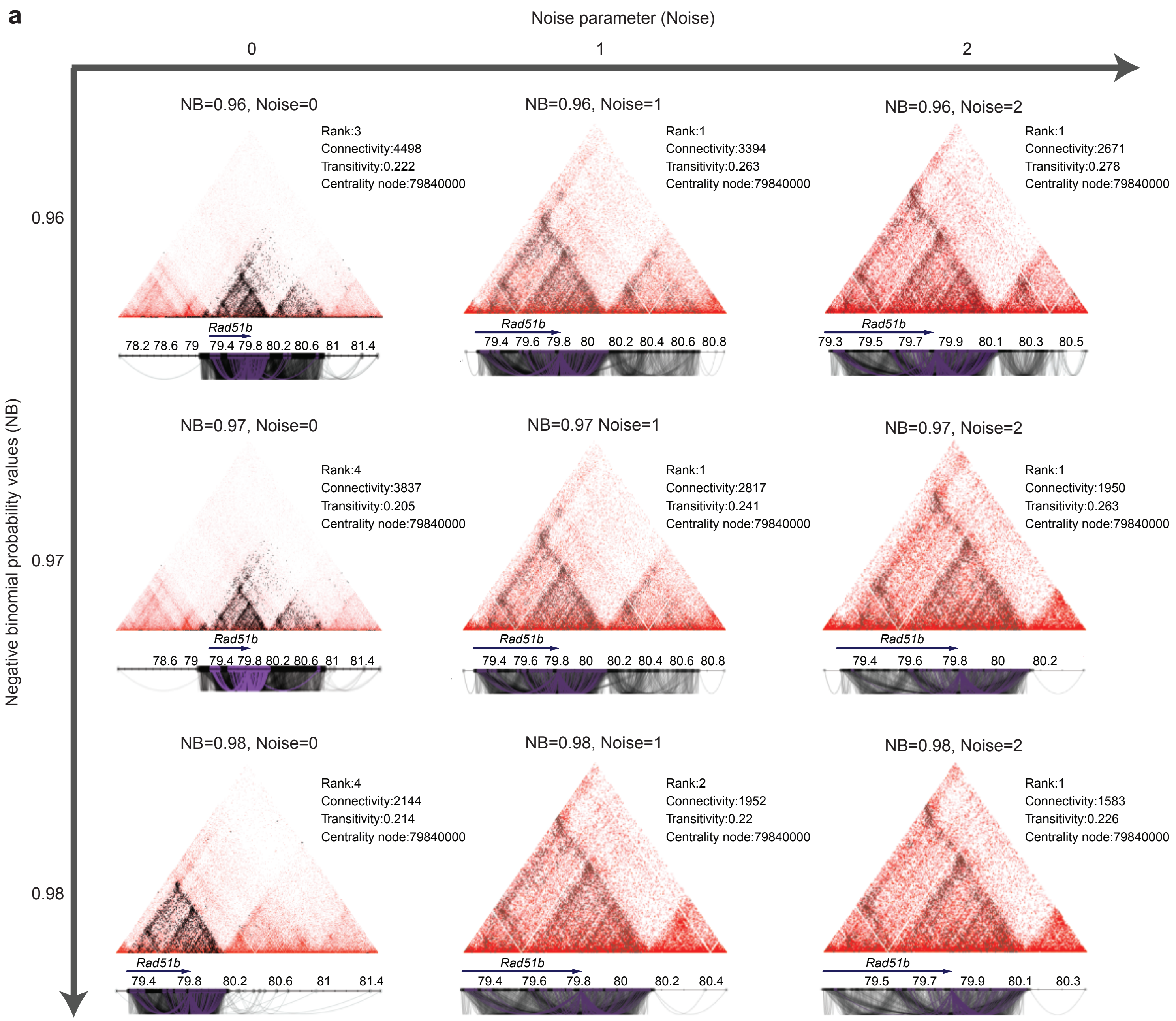

Figure S1. Parameter optimization for Hi-Cociety

(a) To suggest the optimal values for the negative binomial (NB) and noise parameters for defining chromatin modules, we tested various parameter combinations using Hi-C data with a fixed number of valid pairs. Specifically, we downsampled deeply sequenced large pre-B cell Hi-C data[6] to 2 million valid pairs and evaluated module formation at the Rad51b locus under nine parameter combinations (NB = 0.96, 0.97, 0.98; noise = 0, 1, 2). As expected, higher NB values and stricter noise cutoffs led to the identification of denser modules. Notably, the difference between noise settings 0 and 1 was substantial, highlighting the critical importance of filtering out noisy chromatin interactions.

bioRxiv preprint doi: https://doi.org/10.1101/2025.06.12.659372; this version posted June 17, 2025. The copyright holder for this preprint (which was not certified by peer review) is the author/funder, who has granted bioRxiv a license to display the preprint in perpetuity. It is made available under aCC-BY 4.0 International license.

Figure S2

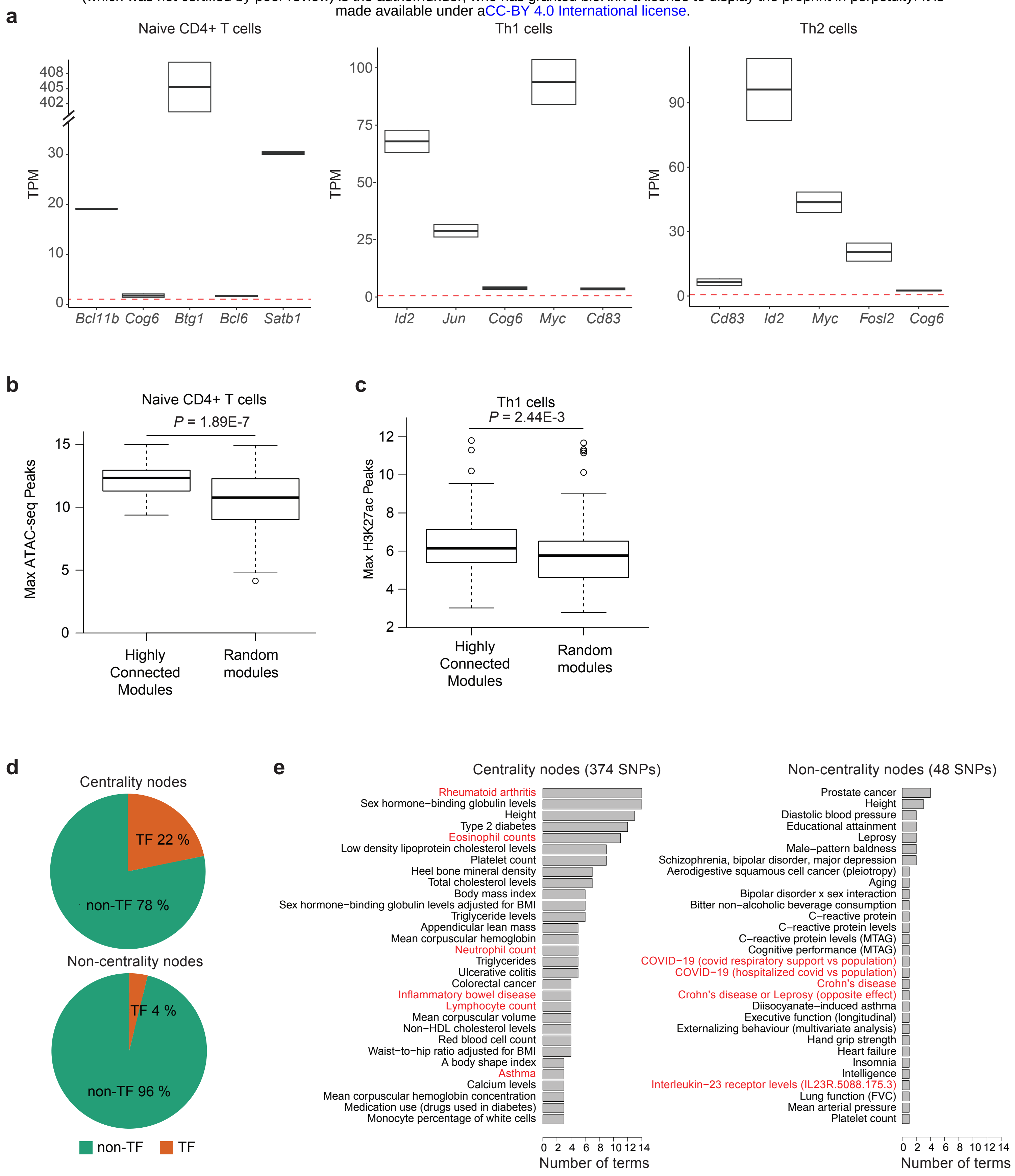

Figure S2. Connectivity of modules correlates with transcriptional and epigenetic activity
(a) Expression levels of representative genes from the five most highly connected modules in each of three T cell subtypes. The dashed red line indicates the median expression level of all genes with TPM > 0.

(b–c) Comparisons of (b) maximum chromatin accessibility (ATAC-seq peaks) and (c) maximum histone acetylation (H3K27ac) levels between the 100 most highly connected modules and 100 randomly selected modules.

(d) In Th1 cells, 22% of central nodes in the top 100 modules encode transcription factors, compared to only 4% of non-central nodes.

(e) Central nodes harbor a greater number of SNPs associated with immune diseases compared to the least central nodes.

bioRxiv preprint doi: https://doi.org/10.1101/2025.06.12.659372; this version posted June 17, 2025. The copyright holder for this preprint (which was not certified by peer review) is the author/funder, who has granted bioRxiv a license to display the preprint in perpetuity. It is made available under aCC-BY 4.0 International license.

Figure S3

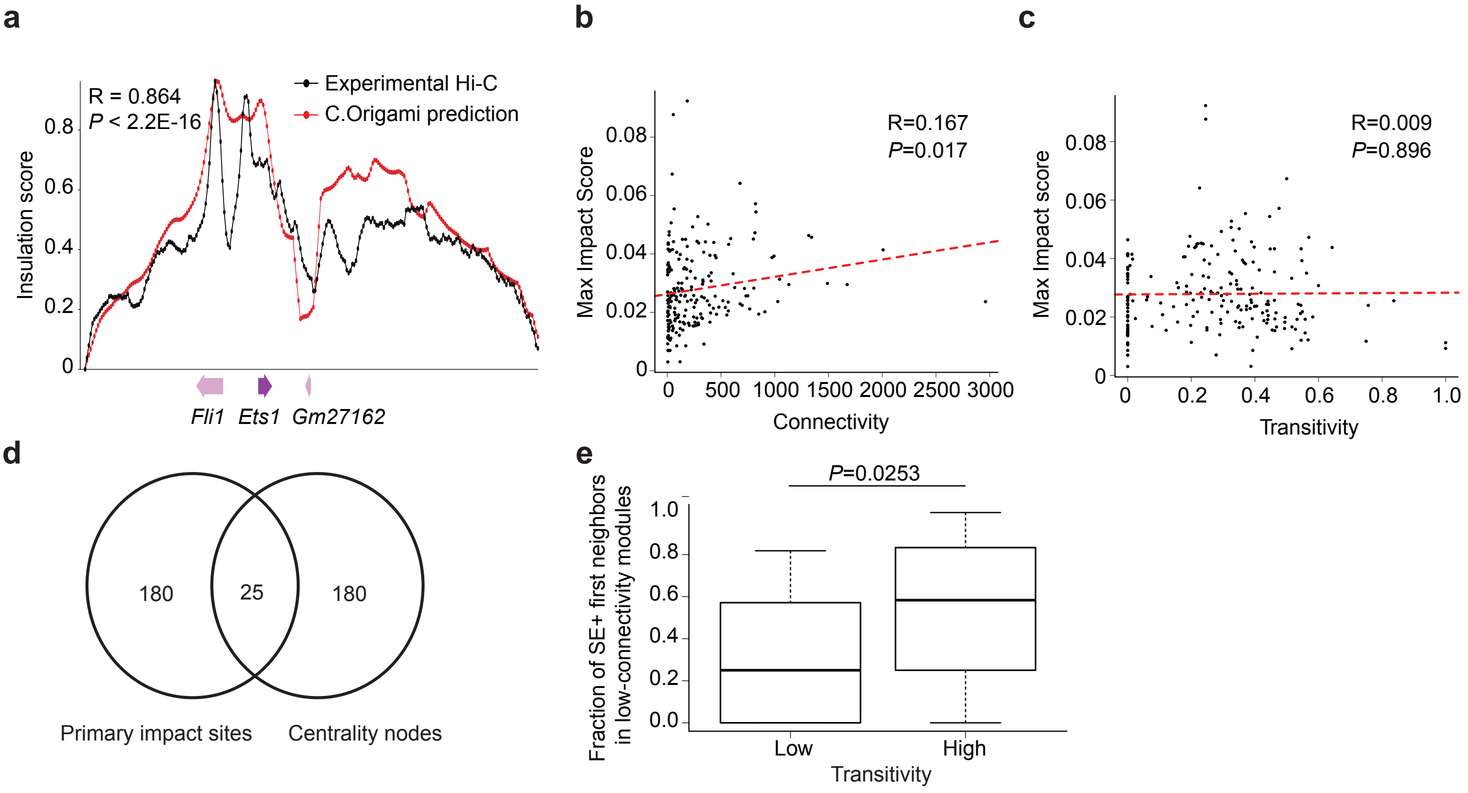

Figure S3. Validation of C.Origami predictions through in silico perturbation analysis (a) Insulation scores from experimental Hi-C data of *Gm27162*-deleted naïve $CD4^+$ T cells strongly correlate with C.Origami predictions at the *Fli1-Ets1* locus (R = 0.864).

(b–c) Relationships between maximum impact score and (b) connectivity and (c) transitivity among modules on chromosome 2 in naïve $CD4^+$ T cells.

(d) Overlap between primary impact sites identified by C.Origami and central nodes in each module.

(e) For lower-connectivity modules containing super-enhancer, the fraction of first-neighbor nodes that also harbor super-enhancers was compared between modules with high and low transitivity.
